# A One-Step, Triplex, Real-Time RT-PCR Assay for the Simultaneous Detection of Enterovirus 71, Coxsackie A16 and Pan-Enterovirus in a Single Tube

**DOI:** 10.1371/journal.pone.0102724

**Published:** 2014-07-16

**Authors:** Shiyin Zhang, Jin Wang, Qiang Yan, Shuizhen He, Wenbin Zhou, Shengxiang Ge, Ningshao Xia

**Affiliations:** 1 National Institute of Diagnostics and Vaccine Development in Infectious Disease, School of Life Science, Xiamen University, Xiamen, Fujian, China; 2 Xiamen Innovax Biotech Co., LTD, Xiamen, Fujian, China; 3 Xiamen Center for Disease Control and Prevention, Fujian, China; 4 School of Public Health, Xiamen University, Xiamen, China; University Hospital San Giovanni Battista di Torino, Italy

## Abstract

The recent, ongoing epidemic of hand, foot, and mouth disease (HFMD), which is caused by enterovirus infection, has affected millions of children and resulted in thousands of deaths in China. Enterovirus 71 (EV71) and coxsackie A16 (CA16) are the two major distinct pathogens for HFMD. However, EV71 is more commonly associated with neurologic complications and even fatalities. Therefore, simultaneously detecting and differentiating EV71 and CA16 specifically from other enteroviruses for diagnosing HFMD is important. Here, we developed a one-step, triplex, real-time RT-PCR assay for the simultaneous detection of EV71, CA16, and pan-enterovirus (EVs) in a single tube with an internal amplification control. The detection results for the serially diluted viruses indicate that the lower limit of detection for this assay is 0.001–0.04 TCID_50_/ml, 0.02 TCID_50_/ml, and 0.001 TCID_50_/ml for EVs, EV71, and CA16, respectively. After evaluating known HFMD virus stocks of 17 strains of 16 different serotypes, this assay showed a favorable detection spectrum and no obvious cross-reactivity. The results for 141 clinical throat swabs from HFMD-suspected patients demonstrated sensitivities of 98.4%, 98.7%, and 100% for EVs, EV71, and CA16, respectively, and 100% specificity for each virus. The application of this one-step, triplex, real-time RT-PCR assay in clinical units will contribute to HFMD surveillance and help to identify causative pathogen in patients with suspected HFMD.

## Introduction

Hand, foot, and mouth disease (HFMD) is a human syndrome that includes fever; vesicular eruptions on the hands, feet, and the anterior part of the buccal mucosa; and is caused by human enteroviruses in the *Picornaviridae* family [1]–[4]. HFMD usually affects children <5 years of age and has emerged as a significant public health issue in China in recent years [5], [6]. In 2011 and 2012, a total of 3,788,473 cases of HFMD were recorded in China, accounting for 1,076 deaths, according to the Ministry of Health of the People’s Republic of China (http://www.moh.gov.cn/zhuzhan/yqxx/201304/b540269c8e5141e6bb2d00ca539bb9f7.shtml).

The most common etiologic agents for HFMD are enterovirus 71 (EV71) and coxsackie A16 (CA16) [3], [4], [7], [8], [9]–[11]. Although these two viruses are clinically indistinguishable regarding HFMD symptoms [12], EV71 infection is associated more frequently with severe neurologic diseases, such as aseptic meningitis and brainstem and cerebellar encephalitis, which are rarely observed with CA16 infection [3]–[7], [12]. In addition to the above viruses, other enteroviruses, such as coxsackie A4, coxsackie A6, coxsackie A10, coxsackie A12, coxsackie B3, coxsackie B5, echovirus 4, echovirus 19 and echovirus 30, can also lead to HFMD, but generally, patients present with mild symptoms that resolve spontaneously within 2 weeks [3]–[5], [7], [9]. So EV71 infected patients should be given more and longer attention than other HFMD patients in clinical treatment. Thus, simultaneously detecting and differentiating EV71 and CA16 specifically from other enteroviruses is important when diagnosing HFMD [3], [4]. Differentiating EV71 and CA16 specifically from other enteroviruses mainly depends on virus isolation and serotyping [3]. However, this method is not only insensitive but also labor-intensive and time-consuming [2]. Furthermore, some enteroviruses replicate poorly in cell culture [8].

In recent years, reverse transcription-PCR (RT-PCR), with its high sensitivity, specificity and short-window period, has been widely used for detecting enterovirus infection. The traditional method using agarose or polyacrylamide gels to detect PCR products is not suitable for diagnosing HFMD in clinical units because of the complex techniques, time consumption, inconvenience, and insufficient sensitivity [1], [5], [7,]. Advanced real-time RT-PCR techniques eliminate the issues above. However, monoplex real-time RT-PCR [7], [13]–[16] and isothermal amplification [10], which target only EV71, CA16, or pan-enterovirus (EVs), have to be performed 3 times to determine whether an enterovirus infection is present and to identify whether the pathogen is EV71 or CA16. These techniques are inefficient. Therefore, the multiple detection method has become a research hotspot for diagnosing HFMD. There are a number of reports based on multiple real-time RT-PCR methods, i.e., duplex PCR targeting EV71 and CA16 or EV71 and EVs [2], [3], [7], [8]. Although this duplex PCR method can reduce a certain workload, the method is still insufficient due to the need for the simultaneous detection and differentiation of EV71 and CA16 from other enteroviruses. Although the more advanced GeXP assay can identify several serotypes of HFMD-associated enteroviruses, including EV71 and CA16, the assays require nearly 6 hrs for amplification, capillary electrophoresis, and fragment analysis [17]. In addition to the long time that is required, the expensive GeXP system is not as common as the equipment for real-time PCR in clinical units.

In this study, we have established a one-step, triplex, real-time RT-PCR assay (triplex RT-PCR) for the simultaneous detection of EV71, CA16 and EVs in a single tube with a non-competitive internal control (IC). The IC is designed for excluding the false negative results generated by impurities from extract process or other abnormities in amplification. After evaluating control virus cultures and clinical samples, the assay exhibits a favorable sensitivity and specificity. With the ongoing spread of HFMD, using this assay in clinical units will contribute to HFMD surveillance and help identify the pathogen in patients with suspected HFMD.

## Materials and Methods

### Ethics statement

Independent ethics committee approval was obtained from the Ethics Committee of the National Institute of Diagnostics and Vaccine Development in Infectious Diseases (NIDVD). All guardians provided written consent on the behalf of the children participants involved in this study.

### Virus stocks

The HFMD virus stocks include six strains of EV71 (genotype: A, B4, B5, C2, C4, C5), two strains of CA16 (genotype: B1) and nine stains of other enteroviruses (genotype: CA2, CA6, CA9, CA10, CB2, CB3, CB5, E3, Echo30). All of the viruses were cultured in RD or Hep-2 cells (conserved by NIDVD) and titrated using a 50% tissue culture infective dose (TCID_50_) assay.

### Clinical samples

A total of 141 throat swabs were collected, mainly 1–4 daily after symptom onset, from patients with suspected HFMD who visited the hospital and were then submitted to the Xiamen Center for Disease Control and Prevention for identification. The swabs were placed in virus transport medium and maintained at −80°C.

### Primer and probe design

The available sequences for the enterovirus gene from the National Center for Biotechnology Information (NCBI) database (http://www.ncbi.nlm.nih.gov/) were retrieved and aligned. The highly conserved regions in the VP1 sequences were utilized to design primers and probes for EV71 and CA16 using the Primer3 software (http://www.simgene.com/Primer3) and the 5′ untranslated region (5′UTR) for Evs. A comparison of the primer and probe sequences with those in the nucleotide collection database at the NCBI showed no cross-reactivity between the primer set and common human pathogens and the ability to amplify the respective targets. The sequences and details for the primers and probes are listed in Table 1.

**Table 1:** Primers and probes designed for the specific amplification of EV71, CA16 and QEV.

Primer/Probe	Sequence (5′-3′)	Domain
EV71		
F1	TTCATGTCACCYGCGAGYGC	VP1
R1	GCYCCRTATTCAAGRTCTTTCTC	VP1
P1	**ROX**-TAYGACGGRTAYCCCACRTTYGGWGA-**BHQ1**	VP1
CA16		
F2	CAAGTAYTACCTACRGCTGCCAA	VP1
R2	CAACACACATCTMGTCTCAATGAG	VP1
P2	**CY5**- TACCAGCACTRCAAGCYGCGGAG-**BHQ1**	VP1
Evs		
F3	TACTTTGGGTGTCCGTGTTT	5′UTR
R3	TGGCCAATCCAATAGCTATATG	5′UTR
P3	**FAM**- AYTGGCTGCTTATGGTGACRAT-**BHQ1**	5′UTR
IC		
F4	GTCAAGATCCTCAAAGATACAGCT	
R4	ACTCTTGGCCGTTGGTTTG	
P4	**HEX**-AGTTTGGAGTCTTGGATGTCGCAT-**BHQ1**	
IC4^a^	CGTCAAGATCCTCAAAGATACAGCTGC TATTGACCTTGAAACCCGTCAAAAGTTT GGAGTCTTGGATGTCGCATCTAGGAAGT GGTTGATCAAACCAACGGCCAAGAGTCATG	

a IC4 is the sequence for IC.

### IC design

The IC nucleic acids contained primer-binding regions that were designed according to the sequence of the tobacco mosaic virus (isolate Guangyuan, complete genome, http://www.ncbi.nlm.nih.gov/nuccore/HE818460.1). The IC sequence and the details for its primers and probes are listed in Table 1. In order to check from RNA extraction to amplification, IC DNA sequence was inserted into pET28a (+) – MS2 vector and then be constructed to an IC sequence RNA contained armored virus (Wantai, Beijing, China).

### RNA extraction

The viral RNA was extracted from mixture of 120 µL of each clinical sample and 20 µL of above armored virus, and was finally eluted with 60 µL of elution buffer using the QIAamp Mini viral RNA Extraction Kit (Qiagen, Inc. Hilden, Germany), according to the manufacturer’ s instructions.

### Triplex RT-PCR

The triplex RT-PCR reaction was performed in a 50 µL of total reaction mixture containing 0.5 µL of 10 µM each primer (Sangon, Shanghai, China), 0.2 µM of the probe (Sangon, Shanghai, China), 3.2 mM of dNTP (Takara Bio Inc., Japan), 4 µL of 10× buffer (Mg^2+^ plus), 1 U of Taq HS DNA polymerase (Takara Bio Inc., Japan), 0.4 U of AMV Reverse Transcriptase (Promega, Inc.) and 5 µL of extracted RNA template. An optimized protocol for the CFX96 Real-Time PCR Detection System (Bio-Rad Inc., USA) was used as follows: 15 min of reverse transcription at 50°C; 10 min of denaturation at 95°C; 40 cycles of 95°C for 15 sec, and 55°C for 45 sec.

### Nest RT-PCR

As the standard for comparison, nest RT-PCR (nRT-PCR) was conduct according to our previously reported method [4]. Briefly, the specimens were first examined using nRT-PCR and sets of broad-spectrum primers for EVs-5′ UTR and two sets of specific primers for EV71-VP1 and CA16-VP1. Based on the initial sequence analysis of the 5′-UTR, the samples that were positive in the 5′-UTR but negative for EV71-VP1 and CA16-VP1 were then amplified by additional serotype-specific primers for the VP1 region. Finally, all of the amplicons for the VP1 regions were subjected to further sequencing and genotyping.

## Results

### Simultaneous detection of EV71, CA16 and EVs with an IC in a single tube

**Figure 1:**
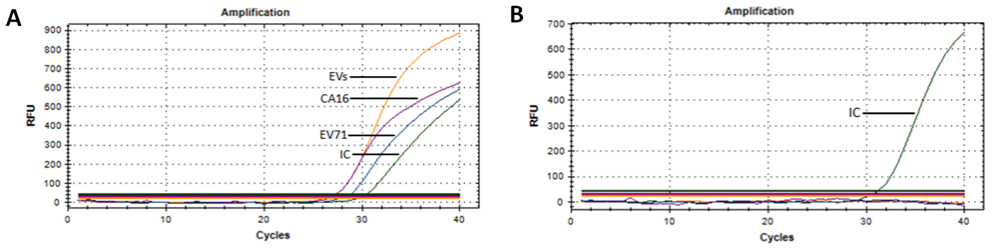
The results from triplex RT-PCR. A. Detection of mixed samples of EV71 and CA16 in one tube. B. Detection of the negative control.

The triplex RT-PCR was developed and optimized using a mixture of EV71, CA16, EVs and IC primers and their corresponding probes. To evaluate the ability to simultaneously detect different pathogens of HFMD, an EV71-C4 virus strain (JS06-52-3) and a CA16-B1 virus strain (3927) at the same concentration of 1 TCID_50_/ml were mixed, and the mixture was then analyzed using triplex RT-PCR. Specific fluorescence curves for ROX (EV71) and CY5 (CA16) were generated from JS06-52-3 and 3927, respectively. Meanwhile, the FAM curve for EVs was generated from the mixture (Fig. 1A). The HEX fluorescence curves for IC, which can demonstrate test validity, were generated in a tube with mixed samples of JS06-52-3 and 3927 (Fig. 1A) and a negative control tube (Fig. 1B).

### Lower limit of detection

**Figure 2:**
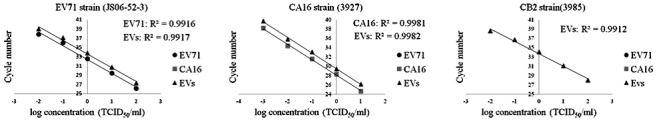
Quantitative analysis of the triplex RT-PCR for detecting EV71, CA16 and EVs- CB2.

Ten-fold serially dilutions of the EV71-C4 (JS06-52-3, 100–0.01 TCID_50_/ml), CA16-B1 (3927, 10–0.001 TCID_50_/ml) and CB2 virus (3985, 100–0.01 TCID_50_/ml) were quantitatively analyzed using 3 channels of triplex RT-PCR, which showed a high correlation coefficient between the Ct value and concentration (JS06-52-3 in the EV71 detection channel: R^2^ = 0.9916; JS06-52-3 in the EVs detection channel: R^2^ = 0.9917; 3927 in the CA16 detection channel: R^2^ = 0.9981; 3927 in the EVs detection channel: R^2^ = 0.9982; 3985 in the EVs detection channel: R^2^ = 0.9912) (Fig. 2).

Based on the above data, the 3 strains were re-diluted in a 2-fold series, 0.08–0.005 TCID_50_/ml for JS06-52-3 and 3985, 0.004–0.00025 TCID_50_/ml for 3927. All of the strains were tested using 20 replicates to determine the lower limit of detection (LoD) for the triplex RT-PCR. The end-point dilution was set at the concentration at which a positive amplification signal was obtained from at least 19 replicates (95%). The variation coefficients for the Ct values for each concentration ranged from 0.1 to 2.58%. The result demonstrated that LoD for the EVs channel for JS06-52-3, 3927 and 3985 were 0.04, 0.001 and 0.04 TCID_50_/ml, respectively, and for the EV71 channel for JS06-52-3 and CA16 channel for 3927 were 0.02 and 0.001 TCID_50_/ml, respectively. As the standard for comparison, the LoD for the EVs nRT-PCR for detecting JS06-52-3, 3927 and 3985 were the same at 0.01 TCID_50_/ml, and for the EV71 nRT-PCR for detecting JS06-52-3 and CA16 nRT-PCR for detecting 3927 were 0.01 and 0.001 TCID_50_/ml, respectively.

### Detection of the virus stocks

To evaluate the ability of the triplex RT-PCR to detect and distinguish different serotypes of EVs, RNA templates extracted from the HFMD virus stocks with a concentration of 0.1 TCID_50_/ml were analyzed using triplex PCR. All 17 strains, including EV71, CA16, CA2, and CA6, etc., tested positive in the EVs detection channel; six strains of EV71 were positive in the EVs and EV71 detection channels and negative in the CA16 detection channel; two strains of CA16 were positive in the EV and CA16 detection channels and negative in the EV71 detection channel (Table 2). Extremely high virus loads for all the 17 strains with titers were between 10^4^–10^5^TCID_50_/mL were tested using triplex PCR, and the results further indicate that there is no cross-reactivity between the EV71 and CA16 detection channels.

**Table 2:** Detection results for the HFMD viral stocks.

Viral stain	Genotype	Detect channels	Viral stain	Genotype
		EV71	CA16	EVs
Vero C4	EV71-A	+	−	+
2006-02203	EV71-B4	+	−	+
2008-03315	EV71-B5	+	−	+
2008-03149	EV71-C2	+	−	+
JS06-52-3	EV71-C4	+	−	+
2008-02969	EV71-C5	+	−	+
3927	CA16-B1	−	+	+
3204	CA16-B1	−	+	+
2008-03352	CA2	−	−	+
2007-00141	CA6	−	−	+
4629	CA9	−	−	+
3160	CA10	−	−	+
3985	CB2	−	−	+
2035	CB3	−	−	+
5428	CB5	−	−	+
3448	E3	−	−	+
4037	Echo30	−	−	+

### Analysis of the clinical samples

A total of 141 throat swabs collected from patients clinically diagnosed with HFMD from March to June 2013 were tested by the triplex RT-PCR. Nest RT-PCRs were tested in the parallel, and the genotypes were identified using our previously reported method [4].

Of the 141 samples, the number of positive samples was 126, 78, and 9 for the EVs, EV71, and CA16 channels, respectively, using triplex RT-PCR (Table 3). Except for 2 false negatives in the EVs detection channel and 1 false negative in the EV71 detection channel, the results for the other samples were all consistent with the nRT-PCR results. The 3 false negatives are attributed to only 2 EV71 samples. One false negative was missed in the EVs and EV71 detection channels, and the other was only missed in the EVs detection channel. Most of the EV71 strains were successfully amplified; therefore, the false negative results for the EVs and EV71 channels in the triplex RT-PCR may be due to lower detection limits compared to the limits for nRT-PCR reported in reference 4 (0.04 TCID_50_/ml vs 0.01 TCID_50_/ml for EVs and 0.02 TCID_50_/ml vs 0.01 TCID_50_/ml for EV71). In conclusion, the triplex RT-PCR exhibited 98.4%, 98.7%, 100% sensitivities for the EVs, EV71 and CA16 strains, respectively, and 100% specificity for all of the strains based on the results of 141 clinical samples (Table 3).

**Table 3:** The triplex RT-PCR results for clinical samples from HFMD patients.

Genotype	No. of samples	Triplex RT-PCR	Genotype	No. of samples
		Evs	EV71	CA16
Enterovirus				
EV71	79	77	78	0
CA16	9	9	0	9
CA5	1	1	0	0
CA6	34	34	0	0
CA10	3	3	0	0
CB2	1	1	0	0
CB4	1	1	0	0
Negative	13	0	0	0
Sensitivity		98.40%	98.70%	100%
		(126/128)	(78/79)	(9/9)
Specificity		100%	100%	100%
		(13/13)	(62/62)	(132/132)

## Discussion

EV71 and CA16 are the two major pathogens that have caused several epidemics of HFMD in recent years in the Asia-Pacific region, including Japan, Malaysia, Singapore, Vietnam and China. Compared to CA16, EV71 causes more serious neurological complications and even death. Although other enteroviruses can also induce HFMD, they often do not lead to serious consequences. Thus, distinguishing EV71 and CA16 from other causes of HFMD will offer significant benefits to clinical management [5].

The application of traditional immunological methods to HFMD diagnosis in clinical units is hindered by significant time consumption and insufficient sensitivity. Recently, nRT-PCR has been widely employed to detect and identify suspected HFMD cases in laboratories but not in clinical units because of its complex procedures and time consumption. Although the available monoplex real-time PCR method eliminates the electrophoresis process in traditional nRT-PCR, the test still needs to be performed more than one time to confirm whether the pathogen is EV71, CA16, or another enterovirus.

Thus, multiplex PCR was developed because this technique can detect more than one target in each reaction. Because multiplex PCR decreases the workload and cost, this assay is becoming a rapid, convenient screening method for HFMD diagnosis. However, multiplex PCR is not a simple superposition of monoplex PCRs. Developing a multiplex PCR assay without losing sensitivity is more difficult compared to monoplex PCR. There are two reasons for this difficulty. On one hand, numerous sets of primers and probes in one reaction may interfere or inhibit each other. Also, the optimum reaction conditions for different sets of primers and probes are diverse, which is why the available so-called “multiplex PCR” is usually just a duplex PCR that generally targets only EV71 and CA16 or only EV71 and EVs when evaluating for HFMD. A method that can simultaneously detect and distinguish EV71 and CA16 from other enteroviruses in a single reaction is still unavailable. To overcome the above issues, we have designed several sets of primers and probes for EV71, CA16 and EVs. After a simple screening and evaluation using monoplex detection, highly sensitive and specific sets of primers and probes were selected and subsequently subjected to cross combination assessment. The combination of primers and probes with minimum interference was selected for further optimization, including ensuring the quantities of each primer and probe, screening the high performance reaction buffer, constructing an IC without any interference.

The one-step, triplex, real-time RT-PCR assay for diagnosing HFMD can simultaneously detect and differentiate EV71 and CA16 from other enteroviruses in one reaction and shows no interferences among the 3 detection channels. The LoD for the EVs channel for the EV71, CA16 and CB2 virus strains was 0.04, 0.001 and 0.04 TCID_50_/ml, respectively, and the LoD for the EV71 and CA16 channels was 0.02 and 0.001 TCID_50_/ml, respectively. The detection results for the HFMD viral stocks indicate that the triplex PCR can detect all 17 EVs strains with a concentration of 0.1 TCID_50_/ml, including 6 sub genotypes of EV71 and 9 serotypes of other enteroviruses, and showed no cross-reactivity between the EV71 and CA16 detection channels when examining high concentrations of the strains (10^4^–10^5^ TCID_50_/ml). In addition, this new method demonstrated 98.4%, 98.7%, and 100% sensitivities for EVs, EV71 and CA16, respectively, and 100% specificity for each strain based on the results from 141 clinical samples.

In conclusion, by establishing a one-step, triplex, real-time RT-PCR assay, a method for the simultaneous detection and differentiation of EV71 and CA16 from other enteroviruses in a single reaction was achieved. Because this method is simpler and provides faster processing times than conventional approaches, using this assay in clinical units will contribute to HFMD surveillance and help identify the pathogen in patients with suspected HFMD.
